# Microstructure and Mechanical Properties of Fe-36Ni and 304L Dissimilar Alloy Lap Joints by Pulsed Gas Tungsten Arc Welding

**DOI:** 10.3390/ma13184016

**Published:** 2020-09-10

**Authors:** Qian Wang, Junqi Shen, Shengsun Hu, Guancheng Zhao, Jie Zhou

**Affiliations:** 1 Tianjin Key Laboratory of Advanced Joining Technology, Tianjin University, Tianjin 300354, China; wqtju@tju.edu.cn (Q.W.); huss@tju.edu.cn (S.H.); zgclive@tju.edu.cn (G.Z.); zhoujie33244@163.com (J.Z.); 2 School of Materials Science and Engineering, Tianjin University, Tianjin 300354, China

**Keywords:** P-GTAW, dissimilar alloy, grain refinement, mechanical properties

## Abstract

High-quality joining of dissimilar alloys between Fe-36Ni alloy and 304L stainless steel is essential in the manufacturing of LNG tanker. In this study, lap joints of Fe-36Ni and 304L dissimilar alloys were fabricated by a pulsed gas tungsten arc welding (P-GTAW) process. The effects of low-frequency pulse on the appearance, microstructure and mechanical properties of the Fe-36Ni/304L lap joints was investigated. With the increase of frequency, the feature sizes of α (the transition angle of the upper surface of Fe-36Ni to the surface of the weld bead) and R (shortest distance between weld root and weld surface) exhibited downtrend and uptrend, respectively, while La (the maximum weld width of lower sheet) and P (the maximum weld penetration of lower sheet) changed in a smaller range. Fusion zone (FZ) is mainly composed of γ phase and M_23_C_6_ during solidification, and M_23_C_6_ particles are distributed on the grain boundaries of the cells, which reduced the mechanical properties of joint. The average hardness between 110 HV_1_ and 136 HV_1_ is lower than that of the base metals. Fractures of all joints located at the Fe-36Ni side near the weld, and a dimple fracture in all samples indicated a ductile fracture. This study found that the heat input values remain 198.86 J mm^−1^ and increased pulse frequency can improve the maximum tensile force. The average maximum tensile force of the lap weld is 11.95 kN when pulsed frequency is 15 Hz.

## 1. Introduction

In recent years, the scale of the liquid natural gas (LNG) transportation market increased with increased international demand for LNG, which poses new challenges to the design of LNG carrying capacity. The maintenance system of the LNG ship tanker is the most critical part. Its function is to store the LNG with a liquid temperature of −163 °C [1]. Therefore, the materials selected for the maintenance system must be safe in cryogenic environments. Fe-36Ni alloy with face-centered cubic (FCC) crystal structure, famous as invar alloy, has a very low linear expansion coefficient in the temperature range below Curie (230 °C). Fe-36Ni is the perfect insulation material for LNG maintenance system. 304L is an economical austenitic stainless steel with the excellent corrosion resistance and low temperature toughness, which can effectively prevent the occurrence of malignant brittle fracture accidents. Nowadays, Fe-36Ni and 304L have been widely used in building of LNG ships and LNG-related industries [2,3,4,5,6].

Fe-36Ni is austenitic steel, mainly composed of Fe and Ni. The composition and structure of Fe-36Ni were similar to typical nickel-based alloys. Therefore, the welding problem of nickel-based alloy and austenitic stainless steel had guiding significance for this study. Shakil et al. [7], reported that there were a few micro-cracks in the fusion zone during the welding process of 304L and Inconel 625 dissimilar alloys. During the solidification of nickel alloy, Ni, S, and P formed liquid state membrane with low melting points respectively. In the late solidification, liquid state membrane was subjected to tensile stress and thus lead to hot cracks [8,9]. Ni et al. [10], successfully connected invar steel via laser welding technology. It is found that the lap joint had a lower hot crack sensitivity than the butt joint, because the cracked liquid state membrane of the lap joint during solidification can obtain the filler from the upper molten pool metal. Furthermore, there will be brittle phase precipitation in the welding seam due to the different chemical composition of dissimilar metals, which decreased the joint mechanical property [11,12,13,14]. Lee et al. [15], found faster cooling rates could inhibit the precipitation of chromium carbides in the fusion zone when welding 690 alloy and 304L dissimilar alloys. However, as far as the author knows, researchers mainly focused on the relationship between microstructure and mechanical properties when welding dissimilar metals, and often ignore the influence of macro morphology on joint performance. The macro morphology of the weld also had an important influence on the mechanical properties of the weld. Guo et al. [16], found that the tensile fracture load of 301L lapping laser welded joint increased with the increase of weld width. Wang et al. [17], found that the effective connection area was proportional to the maximum tensile force by lapping the 0.7 mm/1.0 mm Fe-36Ni alloy sheets via pulsed gas tungsten arc welding (P-GTAW). P-GTAW can accurately control arc capacity and distribution by adjusting welding parameters of pulse specification, which reduced the influence of heat accumulation of welding parts to control weld forming [18].

P-GTAW had been used to weld dissimilar alloys. Moganraj et al. [19], used continuous current GTAW and pulsed argon tungsten arc welding to achieve the welding of AISI 4340 and AISI 304L. In the tensile performance test, it was found that the 4340/304L dissimilar joint using P-GTAW has better tensile performance. Ramkumar et al. [20], used P-GTAW to achieve the connection of Monel 400 and Hastelloy C276 dissimilar metals. It is found that P-GTAW can reduce the segregation rate of Mo, inhibit the occurrence of intermetallic compounds, and improve the mechanical properties of the joint by controlling the heat input and cooling rate. According to the above, P-GTAW is characterized by precise control of the weld geometry and inhibition of the precipitation of the brittle phase in the weld. Therefore, this research used pulsed tungsten argon arc welding to weld Fe-36Ni and 304L.

In this study, the effects of the pulse frequency on the weld formation of Fe-36Ni/304L lap joints was systematically studied and analyzed the microstructure evolution behavior and mechanical properties of the welded joints.

## 2. Experimental Procedure

Two base materials used in this study were Fe-36Ni and 304L alloys, respectively. The dimensions of Fe-36Ni and 304L alloys were 350 mm × 100 mm × 0.7 mm and 350 mm × 100 mm × 2 mm, respectively. The chemical compositions of the two materials are shown in Table 1.

**Figure 1:**
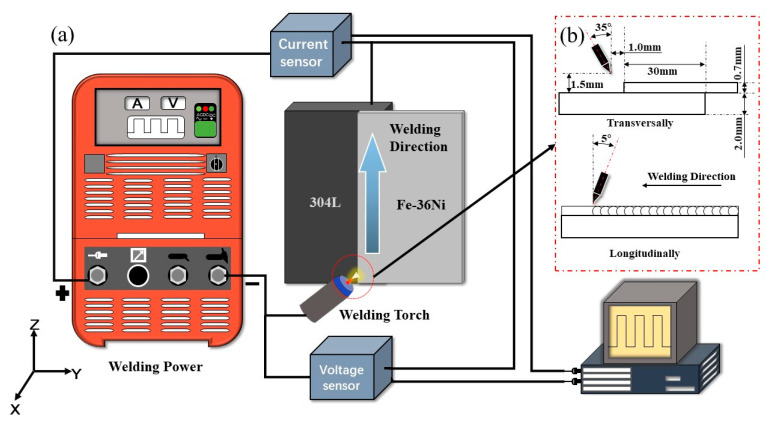
Schematic diagram of (**a**) welding setup and (**b**) position of tungsten electrode.

As shown in Figure 1a, the lap welded joint of dissimilar alloys was fabricated by using the P-GTAW in the vertical upward position. The Fe-36Ni sheet was placed on the 304L sheet (transverse direction), and the lap length was 30 mm. The P-GTAW process was performed by using a Fronius Magic Wave 4000 welding power source (Fronius, Wels, Austria). The tungsten electrode with a diameter of 2.4 mm was positioned as shown in Figure 1b. A Motoman HP6 6-axis robot (Yaskawa Electric, Shanghai, China) was adopted to control the welding speed and path of the GTAW torch. Before welding, the workpieces were cleaned with alcohol to remove the impurities. The welding current and welding voltage were collected by an electric signal acquisition system with a sampling frequency of 10 kHz. The composition of the acquisition system is similar to that in the reference [21]. According to Joseph et al. [22], the heat input (*HI*) can be calculated as follow Equation (1).

(1) $$HI=\frac{\int_{0}^{t}U_{i}I_{i}dt}{t\times v}\times \eta$$

where *U_i_* and *I_i_* are instantaneous voltage and current, respectively, v is the welding speed, *η* is the heat transfer efficiency equal to 70% [23], and *t* is the sampling time. The main P-GTAW welding parameters and calculated heat input for different welding parameters are shown in Table 2.

**Figure 2:**
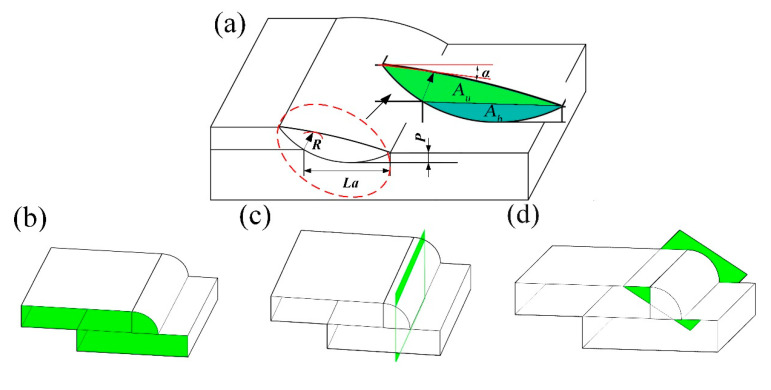
Macroscopical geometry of lap welding section (**a**) measurement of feature sizes and weld cross-sectional area, (**b**) cross section, (**c**) longitudinal section and (**d**) parallel section.

As shown in Figure 2, the metallographic specimens cut from the welded joints in three sections (i.e., cross section, parallel section and longitudinal section) were polished and finally etched by an etchant (5 g FeCl_3_ + 10 mL HNO_3_ + 20 mL H_2_O) for 20 s. Four feature sizes (i.e., *La*, *P*, *R* and *α*) of the lap joint were measured. As shown in Figure 2a, *La*, *P*, *R* and *α* represent the maximum weld width, the maximum weld penetration in the lower sheet, the shortest distance from the weld root to the weld surface, and the transition angle of the upper surface of Fe-36Ni to the surface of the weld bead, respectively.

**Figure 3:**
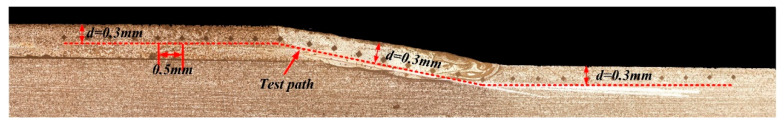
Schematic of hardness test.

**Figure 4:**
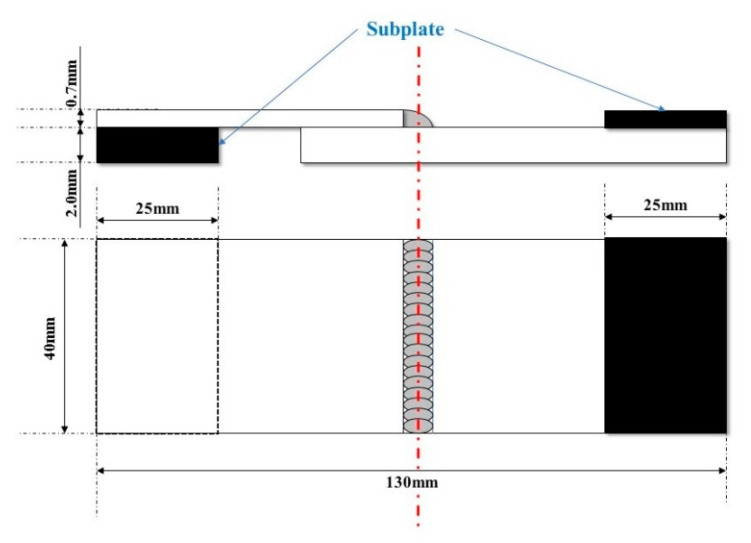
Schematic of tensile sample.

The microstructure was observed using an OLYMPUS GX51 optical microscope (OM) (OLYMPUS, Tokyo, Japan) and Hitachi S-4800 scanning electron microscope (SEM) (Hitachi, Tokyo, Japan) coupled with an energy dispersive spectrometer (EDS) (Hitachi, Tokyo, Japan). X-ray diffraction (XRD) analysis for phase identification was conducted by a D8 ADVANCE X-ray diffractometer (Bruker, Karlsruhe, Germany) with reflection geometry and CuKα radiation (λ = 0.154 nm). The scanning was made through 2θ = 30–100° with a step interval of 0.02°. The phase composition of the weld metal was calculated using Jmatpro software (v 10.0, Sente Software Ltd., Surrey Research Park, Guildford, UK). The hardness test was performed using an HV-1000A micro-hardness tester (Laizhou Huayin Test Instrument Co., Ltd., Yantai, China) with a load of 1000 g for 15 s, and the distribution of test indentations is shown in Figure 3. Tensile tests were conducted by a CSS-44100 universal testing machine (Changchun New Testing Machine Co., Ltd, Changchun, China) with a loading rate of 2 mm/min, and the size of tensile samples is shown Figure 4.

## 3. Results and Discussion

### 3.1. Macro-Morphology of the Welded Joints

**Figure 5:**
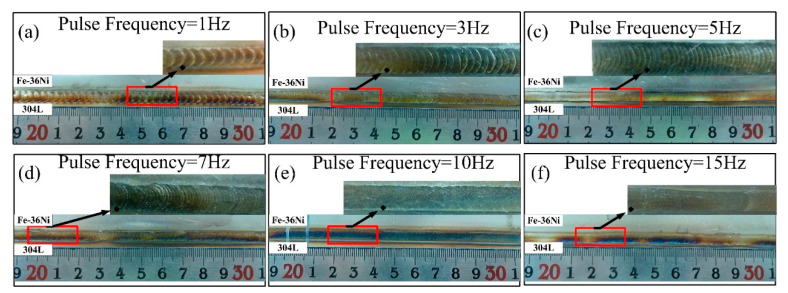
Surface appearance of the weld: (**a**) 1 Hz, (**b**) 3 Hz, (**c**) 5 Hz, (**d**) 7 Hz, (**e**) 10 Hz and (**f**) 15 Hz.

Figure 5 shows the macroscopic morphological characteristics of the lap joints obtained at different pulse frequencies, and the welds showed good appearances without oxidation and visible cracks. The surface of the weld was relatively rough at the frequency of 1 Hz, and it became tighter with the increase in the pulse frequency.

**Figure 6:**
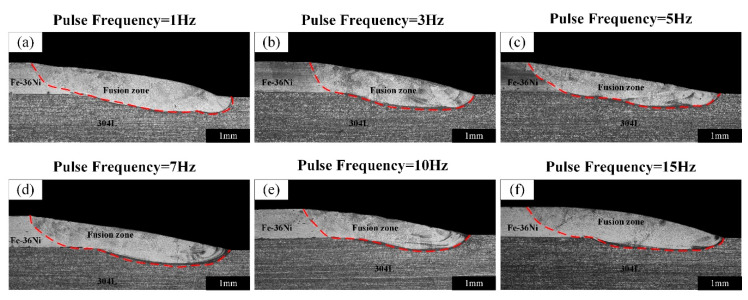
The cross-sectional of the lap joints at different pulse frequencies: (**a**) 1 Hz, (**b**) 3 Hz, (**c**) 5 Hz, (**d**) 7 Hz, (**e**) 10 Hz and (**f**) 15 Hz.

**Figure 7:**
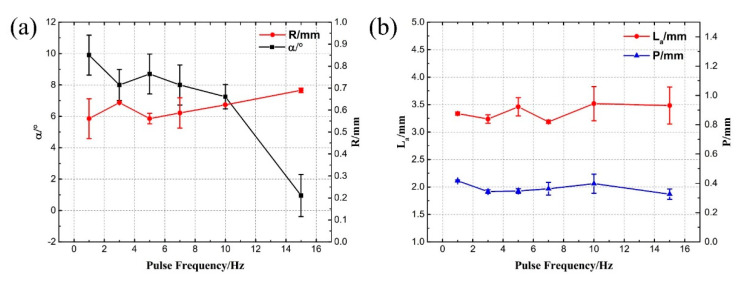
The feature sizes of different pulse frequencies: (**a**) *R* and *α*, (**b**) *La* and *P*.

The cross-sectional metallographic images of the lap joints at different pulse frequencies are shown in Figure 6, and there are no obvious cracks in the welds. As shown in Figure 7a, the feature size *α* decrease with the increase of the pulse frequency and *R* is directly proportional to the pulse frequency. Figure 6f shows that the smooth transition of the weld surface could be obtained when frequency was 15 Hz. Under this condition, *R* was 0.69 mm and *α* was 0.95°. Figure 7b indicates that *La* and *P* changed in a smaller range with the increase of pulse frequency due to the almost constant thermal input at different pulse frequency. The average values of *La* and *P* were 3.37 mm and 0.37 mm, respectively.

**Figure 8:**
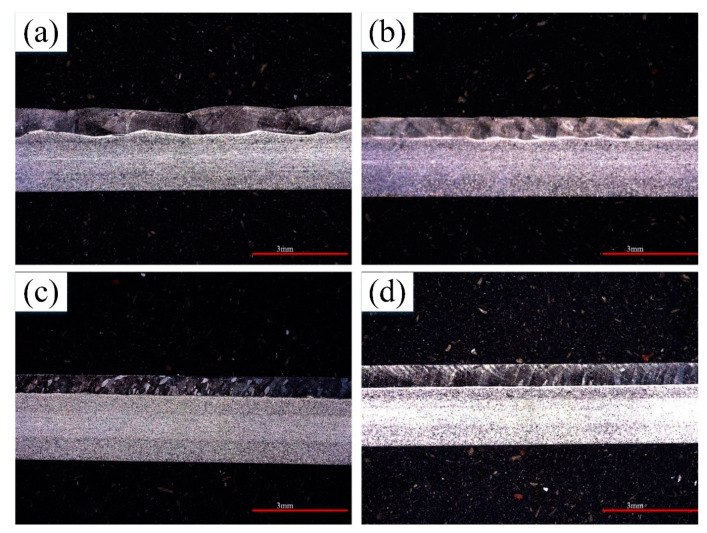
The longitudinal section of the lap joints at different pulse frequencies and schematic of molten pool behavior at different pulse current stages: (**a**)1 Hz, (**b**)3 Hz, (**c**)10 Hz, (**d**)15 Hz.

Figure 8a–d represent longitudinal section of lap joint, the overall weld penetration depth was different when the pulse frequency was less than 5 Hz (Figure 8a,b). At peak current stage and the base current stage, the arc acted positions on the weld surface were different when pulse frequency was smaller. At peak current stage, the center of the molten pool was subjected to the largest plasma pressure and the penetration depth was greater. At base current stage, the plasma pressure was reduced, and the penetration depth was smaller [24]. As the pulse frequency increases, the positions of the peak current and the base current in a unit time gradually approach, and the overall weld penetration difference gradually decreases. When the frequency is greater than 10 Hz, the welding process becomes more stable and the heat acted on the weld is more uniform over the unit length of the weld, resulting in a uniform penetration depth (Figure 8c,d).

**Figure 9:**
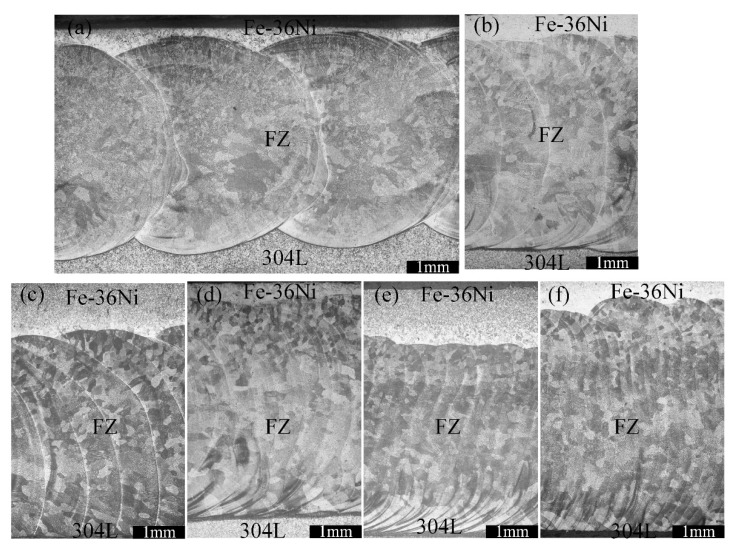
The parallel section of the lap joints at different pulse frequencies: (**a**)1 Hz, (**b**)3 Hz, (**c**)5 Hz, (**d**)7 Hz, (**e**)10 Hz, (**f**)15 Hz.

Figure 9 shows that the parallel section of the lap joints of the weld with different pulse frequencies. Due to the limited dimension of P-GTAW spot, it could be clearly seen that the melting boundary of parallel section was discontinuous and periodical when the pulse frequency was less than 7 Hz in Figure 9a–c. Inconsistent weld morphology will deteriorate joint property, thus avoid the discontinuous and periodic appearance of the melting boundary as much as possible. The parallel section of the lap joints will change according to different pulse frequency. As is shown in Figure 9d–f, continuous weld appearance in the parallel section can be obtained when the pulse frequency was greater than 7 Hz.

### 3.2. Microstructure of the Lap Joints

Zhang et al. [25], studied the laser welding of dissimilar aluminum alloy lap joints. This research found that the chemical composition of the welded joints can be changed by controlling fusion ratio to reduce the thermal cracking sensitivity of the welded joints. In this study, the fusion ratio is used to calculate the chemical composition of the weld (shown in Figure 2a), and the calculation results were input into the Jmatpro software to calculate the phase composition of the weld. The results of chemical composition were shown in Table 3. It is calculated as follows Equations (2) and (3):

(2) $$D\%\mathrm{weld}=\left[\frac{A_{u}}{A_{u}+A_{b}}\right]$$

(3) E%weld=(E%Fe-36Ni)×D%+(E%304L)×(1−D%)

where *A_u_* is the fusion area of the upper sheet, and *A_b_* is the fusion area of the lower sheet, *E%* Fe-36Ni and *E%* 304L are the content of chemical composition in Fe-36Ni and 304L, respectively. The increase or decrease of Cr and Ni content in weld had great influence on the phase composition of weld [26]. The efficiency of welding test can be improved by using the above method to judge the phase composition in the welding seam.

The SEM-EDS results showed that the weld metal at 10 Hz was composed of 1.22 wt% C, 4.58 wt% Cr, 64.42 wt% Fe and 29.78 wt% Ni. By comparing the results in the Table 3, it is found that the accuracy of the calculated chemical composition in the weld metal was high.

**Figure 10:**
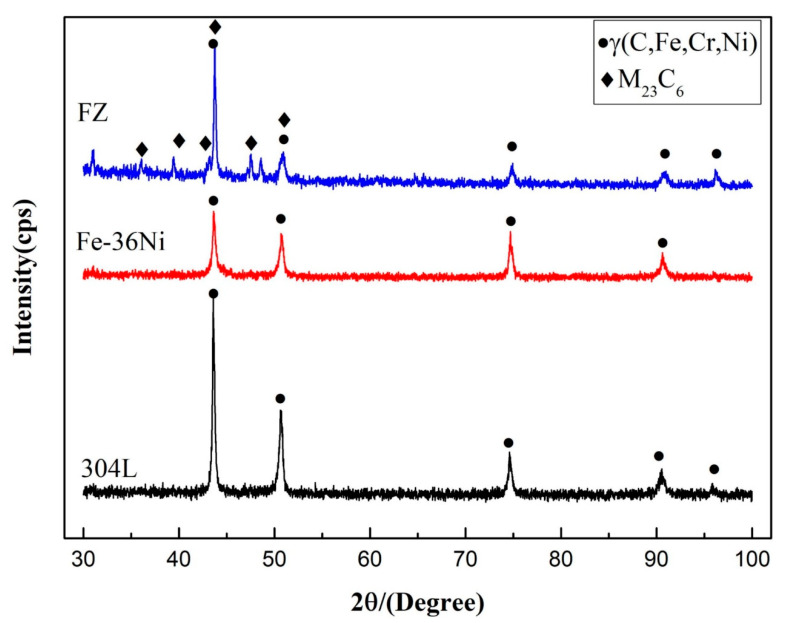
XRD pattern of the lap joint at frequency of 10 Hz.

According to the results in the Table 3, the phase content of the weld metal was calculated via the Jmatpro software. The Jmatpro results displayed that the melting zone was mainly composed of *γ* phase and carbide with the contents of 98.67% and 1.33%, respectively. As shown in Figure 10, the weld metal was mainly composed of austenite and M_23_C_6_, which was consistent with the calculation results based on the Jmatpro software.

**Figure 11:**
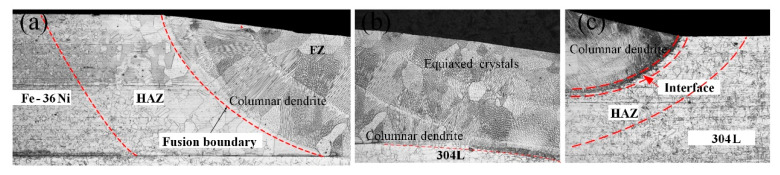
The cross-sections of joints at 10 Hz: (**a**) Fe-36Ni side (**b**) FZ and (**c**) 304L side.

**Figure 12:**
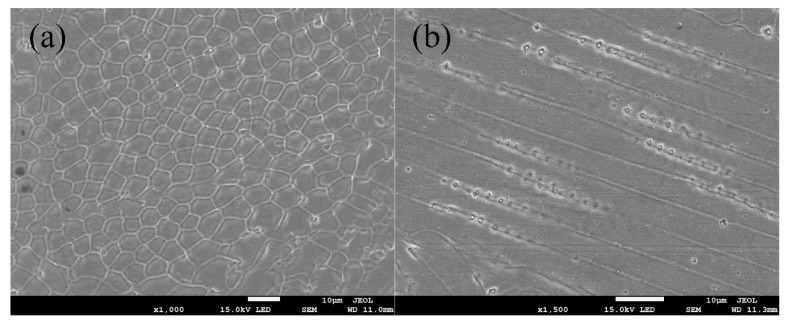
The substructure of FZ: (**a**) Cellular crystal, (**b**) Columnar dendrite.

Figure 11 shows the microstructure of the dissimilar lap joint at 10 Hz, and the fusion zone consisted of austenite. The substructure of the fusion zone showed columnar dendrites and cellular crystal, as shown in Figure 12.

The researchers [27], summarized the effects of temperature gradient *G* and growth rate *R* on the solidification structure of the alloy. It was found that the most important parameter affecting the solidification form was *G/R*. According to Equation (4). When the molten pool is crystallized, the melting boundary preferred starts to crystallize. The solidification mode will move from planar to cellular, cellular and columnar dendrites, and finally to equiaxed crystals with the temperature gradient decreases. As shown in Figure 11a,c, columnar crystals grow towards the weld center at the fusion boundary. In addition, there are equiaxed crystals at the top of the weld as shown in Figure 11b, because the *G* decreases with the increase of distance from the fusion line which resulting in *G* is small in the weld center.

(4) $$\frac{G}{R}\geq \frac{\Delta T}{D_{L}}$$

where *ΔT* is the temperature difference across the interface layer, and *D_L_* is the diffusion coefficient.

As shown in Figure 11a,b, the width of the heat affected zone (HAZ) in Fe-36Ni side was greater than that in 304L side. This is because the thermal conductivity of Fe-36Ni (4.63W m^−1^ K^−1^) is less than that of 304L (16.3W m^−1^ K^−1^). During the welding process, the temperature gradient in the Fe-36Ni side was steeper than that in the 304L side, leading to the obvious grain growth in the HAZ in Fe-36Ni side.

**Figure 13:**
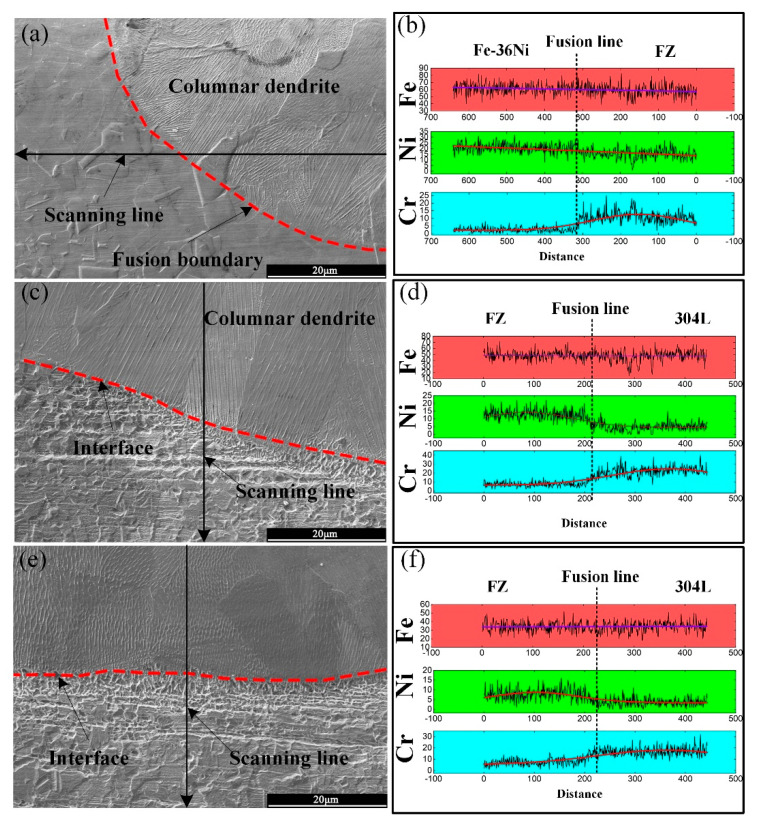
EDS linear analysis near the fusion boundaries at 10 Hz: (**a**) Fe-36Ni alloy side, (**b**) area near the weld root of 304L, (**c**) area near the welding toe of 304L, (**d**) linear elements distribution of Fe-36Ni side, (**e**) linear elements distribution of area near the weld root of 304L and (**f**) linear elements distribution of area near the welding toe of 304L.

Figure 13 is a low magnification SEM micrograph at the fusion boundaries. There was epitaxial growth near the fusion boundary on the Fe-36Ni side, and new grains nucleated at the fusion boundary. The composition of the weld metal on the 304L side was quite different from that of the base metal, so the weld metal was crystallized in a non-epitaxial manner at the melting boundary. There is a transition zone at the interface between 304L and FZ. Ding et al. [28], studied the microstructure of 9%Cr/CrMoV dissimilar welded joints. It was found that the transition zone was caused by the difference in the content of Cr element between the FZ and 9%Cr base materials.

Because Fe-36Ni and 304L have obvious differences in composition, and the composition of the FZ is also different from any other base material. The Ni content and Cr content in the Fe-36Ni alloy 304L are relatively high, respectively, The Ni and Cr contents showed a significant change at the fusion boundary. Figure 13b,d,f shows the liner EDS results of Fe, Cr and Ni elements at the three positions of lap joint. In area of Fe-36Ni to FZ, the Cr content changes greatly, and no transition layer appears. In area of FZ to 304L, an analysis of the EDS linear distribution showed similar characteristics, Cr content increased, and Ni content decreased.

**Figure 14:**
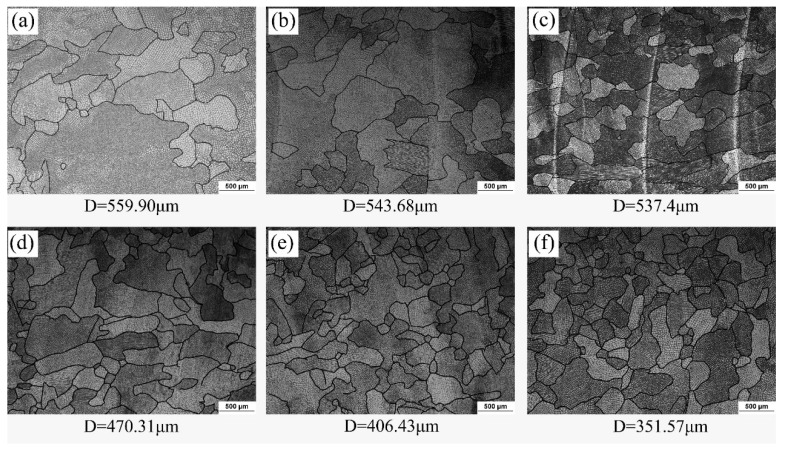
Metallographic of weld center of parallel section at different pulse frequencies: (**a**) 1 Hz, (**b**) 3 Hz, (**c**) 5 Hz, (**d**) 7 Hz, (**e**)10 Hz, (**f**)15 Hz.

The metallographic photographs of the weld center in the parallel section are shown in Figure 14 and all the FZs were composed of equiaxial crystals. Image software was used to measure the average grain size, and the results showed that the average grain size decreased with the increase of pulse frequency. The reasons for the grain refinement caused by increasing the pulse frequency are usually as follows. With the pulse frequency increased, the numbers of the plasma flow force on the molten pool per unit time increased, which caused strong electromagnetic stirring in the molten pool, breaking the dendrites and increasing the nucleation sites, and eventually increasing the grain numbers in the FZ [29,30,31]. But this does not mean that simply increasing the pulse frequency can always refine the grains, and too high welding pulse frequencies will cause the weld structure deterioration. Shao et al. [32], reported that too high a pulse frequency reduced the mechanical properties of the welded joint. Because the effect of the pulse current is too strong in the molten pool at a higher welding frequency. The molten pool cannot form a disordered dendrite arrangement, resulting in the area of equiaxed grains in the center of the weld reduced. In this study, the grain size of the weld center at the pulse frequency of 15 Hz was 35% smaller than that at the pulse frequency of 1 Hz.

**Figure 15:**
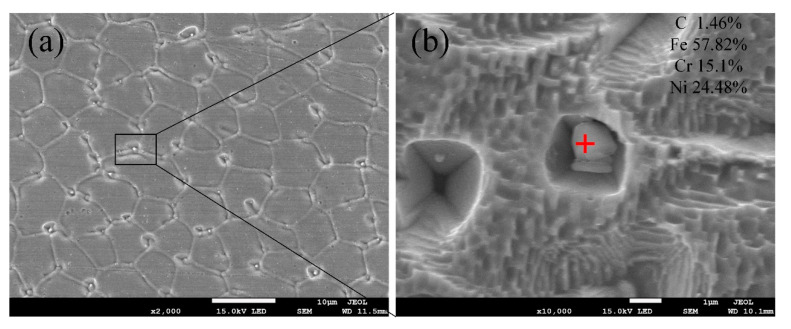
(**a**) Carbides at the grain boundaries and (**b**) High-magnification micrograph of the carbide.

As shown in Figure 15, some particles were distributed at the cellular grain’s boundaries near 304L under SEM observation. The results of EDS analysis indicated that the particle’s main chemical compositions were C, Fe, Cr, and Ni. According to XRD results, the particle should be M_23_C_6_.

Fe-36Ni and 304L have thermal conductivity, and the heat accumulation makes the temperature of the base material gradually increase with the progress of welding [33], which resulting in weld center can maintain a high temperature for a long time. So that the composition fluctuation at the boundary of cell crystal is large enough, and the carbide is enough to nucleate, so that a large number of precipitations occur.

### 3.3. Microhardness

**Figure 16:**
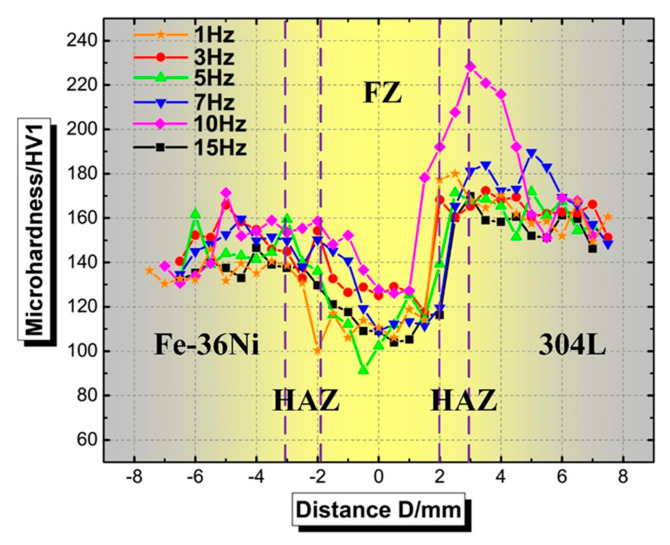
Microhardness distribution of Fe-36Ni/304L lap joints at different pulse frequencies.

Figure 16 indicates that the microhardness of the FZ varied in a small range with the increase of the pulse frequency, and the average microhardness of the FZ at 1 Hz, 3 Hz, 5 Hz, 7 Hz, 10 Hz and 15 Hz was 110 HV_1_, 126 HV_1_, 114 HV_1_, 121 HV_1_, 136 HV_1_ and 112 HV_1_, respectively. The grain size in the heat-affected zone on the Fe-36Ni side is larger and the hardness of this area is lower.

The influence of the HAZ on the mechanical properties of the joint is through the hardening and softening caused by the welding thermal cycle. For the heat-affected zone in the 304L side, the microhardness tends to increase with increasing distance from the center of the weld and reached up to a maximum hardness of 164 HV1. Rogalski et al. [34], studied the mechanical and microstructural of Incoloy 800 HT and austenitic stainless steel 304L dissimilar joints manufactured by gas tungsten arc welding, it is found that the hardness of the HAZ near 304L decreases, which results from the structure transform to δ ferrite. However, Shakil M. et al. [7], studied the welding of 690 alloy and 304L dissimilar alloys via electron beam welded, it is found there doesn’t exist hardness decreases. The hardness test results of this study also did not find a decrease in the hardness on the 304L side. In summary, the main reason for the changes in the mechanical properties of the HAZ near 304L is the δ ferrite transformation caused by thermal cycling. The pulsed tungsten arc welding process has a small heat input and short high temperature residence time, which inhibits δ ferrite transformation.

Although the grains of FZ were refined with increasing the pulse frequency, the grains were still were relatively coarser than those of the parent materials (27.97 μm of Fe-36Ni and 17.23 μm of 304L), resulting in the lowest hardness of the FZ.

### 3.4. Tensile Properties

**Figure 17:**
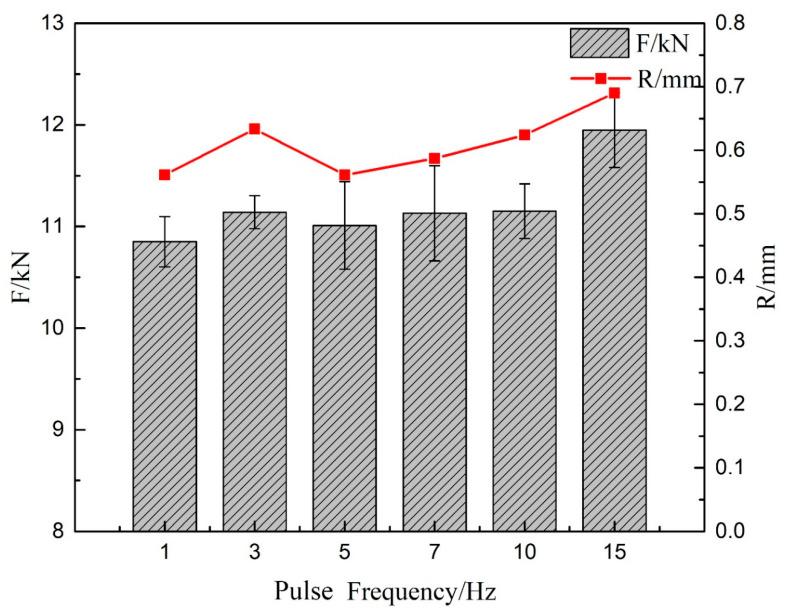
*F* and feature size *R* at different pulse frequencies.

The Table 4 shows the tensile test results for the welded joints obtained at different pulse frequencies. All tensile samples fractured at weld roots, which can be explained for two reasons. On the one hand, the lap joint leads to the stress concentration at the weld root under tensile load. On the other hand, the inhomogeneous distribution of the weld microstructure and the coarse austenite grains leads to the decrease of the joint performance. As shown in Figure 17, the average fracture tensile force (F) increased with increasing the pulse frequency, and the maximum is 11.95 kN when the pulse frequency was 15 Hz. According to relevant reference [35], the strengnth of Fe-36Ni is 430 Mpa (according to the tensile samples’ size used in this study, the Fe-36Ni strength is converted to 12040 kN). When the pulse frequency is 15 Hz, the F of the joint is decreased by 0.7% compared to Fe-36Ni. The increase in R reduced the curvature of the force streamline under the tensile load, resulting in the reduction of the stress concentration [36]. Although the weld grain size decreased with the increase of pulse frequency, the hardness results indicated that the effect of grain size on tensile properties was limited. Figure 17 shows that there is a strong correlation between *R* and *F,* which means that *R* is the main factor determining the maximum tensile force of the lap joint.

**Figure 18:**
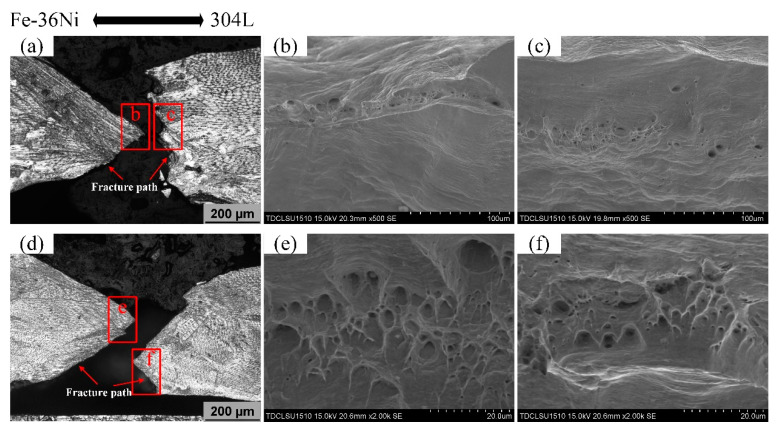
Weld fracture metallography and fracture morphology: (**a**) Fracture cross section at 3 Hz, (**b**) Fracture morphology of the left side of the fracture (low magnification), (**c**) Fracture cross section at 3 Hz, (**d**) Fracture cross section at 10 Hz, (**e**) Fracture morphology of the left side of the fracture (high magnification), (**f**) Fracture morphology of the right side of the fracture (high magnification).

Figure 18 shows the fracture morphologies of the lap joints at 3 Hz and 10 Hz. There are many traces of plastic deformation at the fracture, and sliding and necking areas are obvious, exhibiting a typical plastic fracture as shown in Figure 18a,d. The micro fracture morphology is shown in Figure 18b–f. The elongation of dimples on the two fracture surfaces indicates that the lap joint was subjected to shear force during tensile process. The small fracture zone and sparse dimples on both sides indicate poor ductility of lap joint.

## 4. Conclusions

The pulse tungsten argon arc welding process can be used to connect Fe-36Ni to 304L dissimilar metals. The microstructure and mechanical properties of Fe-36Ni/304L heterogeneous lap joints with different pulse frequencies were studied. Concluded as follow:

(1) There are no visible defects such as void and cracks in the Fe-36Ni/304L lap joint.
(2) The feature size *La* and *P* changed slightly, while *R* showed an increasing trend and *α* showed
(3) A decreasing trend with the increase of pulse frequency.
(4) Fusion zone is mainly composed of *γ* phase and M_23_C_6_, and M_23_C_6_ particles are distributed on the grain boundaries of the cells.
(5) The microhardness of the fusion zone is the lowest in the entire welded joint, with an average hardness between 110 HV_1_ and 136 HV_1_.
(6) The tensile force of Fe-36Ni/304L lap joint has a high positive correlation with the characteristic dimension R. The average maximum tensile force is 11.95 kN when the pulse frequency is 15 Hz.

## Figures and Tables

**Table 1:** Nominal chemical compositions (wt%) of Fe-36Ni alloy and 304L stainless steel.

Element	C	P	S	Si	Mn	Ni	Cr	Fe
Fe-36Ni	≤0.05	≤0.02	≤0.02	≤0.2	0.2–0.6	35.0–37.0	-	Bal
304L	0.03	0.04	0.03	0.75	2	8.0–12.0	18.0–20.0	Bal

**Table 2:** Welding parameters and corresponding heat inputs of the P-GTAW process.

Peak Current/ (A)	Base Current/ (A)	Duty Cycle/ (%)	Pulse Frequency/ (Hz)	Welding Speed/ (mm∙min^−1^)	Gas Flow/ (L∙min^−1^)	Average HI/ (J∙mm^−1^)
100	12	55	1	200	15	199.48
			3			202.75
			5			205.25
			7			201.47
			10			197.52
			15			201.23

**Table 3:** The calculated fusion ratios and chemical compositions of the weld metal (wt%).

Pulse Frequency	C	Ni	Cr	Fe	Si	Mn	D%
1	0.03	27.50	6.89	63.46	0.38	0.80	0.66
3	0.03	27.73	6.73	63.40	0.37	0.78	0.67
5	0.03	27.12	7.15	63.57	0.39	0.82	0.65
7	0.03	28.69	6.06	63.12	0.36	0.72	0.71
10	0.03	28.56	6.15	63.161	0.36	0.73	0.71
15	0.03	30.0	5.09	62.7	0.33	0.63	0.76

**Table 4:** Results of the tensile tests.

Sample	*F* (kN)	Fracture Location
1 Hz	10.85	Weld Root
3 Hz	11.14	Weld Root
5 Hz	11.01	Weld Root
7 Hz	11.13	Weld Root
10 Hz	11.15	Weld Root
15 Hz	11.95	Weld Root
